# Molecular Characteristics of Aberrant Gene Mutations and Expression Profiles Induced by Benzo(a)pyrene in Hepatocellular Carcinoma Cells

**DOI:** 10.3390/toxics12070499

**Published:** 2024-07-09

**Authors:** Xinyi Cao, Ying Zhu, Shujun Cheng, Kunxiao Zhang, Hui Wang, Qian Ba

**Affiliations:** 1 School of Public Health, Shanghai Jiao Tong University School of Medicine, Shanghai 200025, China; 2 Laboratory Center, Shanghai Municipal Hospital of Traditional Chinese Medicine, Shanghai University of Traditional Chinese Medicine, Shanghai 200071, China; 3 Jiangsu Key Laboratory of Marine Pharmaceutical Compound Screening, College of Pharmacy, Jiangsu Ocean University, Lianyungang 222005, China

**Keywords:** BaP, transcriptomic sequencing, exosomal RNA, HCC metastasis

## Abstract

Benzo(a)pyrene (BaP) is a prevalent food and environmental carcinogen. Chronic low-dose BaP exposure can promote the migratory and invasive capacities of human hepatocellular carcinoma (HCC) cells, yet its intricate molecular mechanisms remain elusive. Utilizing the established BaP-exposed HCC cell model, we analyzed the gene expression alteration, exosomal RNA cargo, and genetic variants induced by BaP through transcriptomic and whole-genome sequencing. Transcriptomic analysis revealed significant dysregulation in genes and pathways associated with tumor metastasis, particularly those involved in steroidal lipid metabolism and cell migration. BaP exposure enriched PI3K-AKT, mTOR, and NF-κB signaling pathways and disrupted genes implicated in cellular secretory processes, suggesting the potential involvement of exosomes in metastasis. Exosome analysis depicted the RNA profiling in exosomes of HCC cells altered by BaP, and the exosomal circRNA-miRNA-mRNA interaction network was constructed. Finally, whole-genome sequencing delineated BaP-induced gene mutations and genomic instability in HCC cells. In summary, prolonged low-dose BaP exposure induces intricate molecular alterations in gene mutation and expression profiles in HCC cells, notably those secreted in exosomes, which may potentially remodel the tumor microenvironment and foster HCC metastasis. Our findings offer new insights into the molecular underpinnings of BaP-induced HCC metastasis, thereby advancing the comprehensive understanding of BaP toxicity.

## 1. Introduction

Benzo(a)pyrene (BaP), a characteristic representative of Polycyclic Aromatic Hydrocarbons (PAHs) [1], is ubiquitous in the environment, originating from incomplete combustion of organic components, and can be detected in tobacco smoke, vehicular emissions, fine particulate matter (PM2.5), and certain dietary items [2], especially fried, smoked, and barbecued foods [3]. BaP exposure is almost inevitable, through inhalation and dietary intake [4], and the digestive and respiratory tracts are the main paths of exposure to BaP [5]. Upon entering the body, BaP is distributed to organs such as the liver, mammary glands, and fat through the blood and lymphatic systems [6]. Also, BaP undergoes metabolic activation primarily by the cytochrome P450 enzyme system, yielding reactive metabolites such as BaP dihydroxy epoxide (BPDE), which can form covalent adducts with DNA, leading to the initiation of genomic instability and ultimately contributing to carcinogenesis [7]. Furthermore, BaP metabolism generates reactive oxygen species (ROS), which provoke oxidative stress and harm biomolecules, including nucleic acids, lipids, and proteins [8]. The carcinogenic, teratogenic, neurotoxic, and immunotoxic properties of BaP exposure have been studied [9]. Consequently, the International Agency for Research on Cancer (IARC) has classified BaP as a Group I carcinogen [10], with its carcinogenic potential implicated in various cancers depending on exposure routes [11,12,13]. However, mechanistic investigations often focus on the toxicity endpoints, such as tumor formation, rather than exploring the intricate molecular events underlying BaP-induced toxicity.

The liver plays a crucial role in BaP metabolism and is the major target organ of BaP toxicity. Exposure to environmental BaP is strongly linked to a higher risk of hepatocellular carcinoma (HCC) [14,15]. In terms of tumor progression, our previous research has provided compelling evidence that prolonged low-dose BaP exposure can enhance HCC metastasis [16]. Although there are many studies on the toxic effects of BaP on the liver, the precise molecular mechanisms driving BaP-induced hepatocarcinogenesis remain elusive. To reveal the intricate molecular mechanisms underlying the promotion of HCC metastasis by long-term and low-dose BaP exposure, we hypothesize that BaP induces alterations in gene expression patterns, particularly within exosomal RNA profiles, and modulates crucial cellular processes implicated in HCC progression.

In the present study, we employed the long-term and low-dose BaP exposed HCC cell model and multiple sequencing technologies to explore the molecular behaviors, including the gene expressions and mutations underpinning long-term, low-dose BaP’s toxicity on HCC. Our findings offer novel insights into the molecular mechanisms driving BaP-induced HCC progression and provide a foundation for future research aimed at mitigating BaP-associated health hazards.

## 2. Materials and Methods

### 2.1. Cell Cultures and BaP Exposure

The BEL-7404 cell line was cultured in RPMI 1640 complete medium and sourced from the Chinese Academy of Sciences. Incubation took place at 37 °C within a humidified 5% CO_2_ environment. As reported in our prior research, to explore the potential chronic toxicity of BaP, we developed a low-dose, long-term BaP exposure model. BEL-7404 was co-cultured with different concentrations of BaP (0.01 nM, 1 nM, and 100 nM) or 0.1% DMSO for up to one month (30 days), which then exhibited increased migration and invasion ability even after Bap withdrawal [17]. Therefore, in this study, we used the control and exposed cells (BEL-7404 100 nM) as the experimental model.

### 2.2. RNA Quantification and Quality Assessment

The use of 1% agarose gel electrophoresis allowed for the evaluation of RNA contamination and degradation. A Qubit RNA Assay Kit was utilized to quantify the concentration of RNA, and a NanoPhotometer was utilized to determine its purity. Additionally, an RNA Nano 6000 Kit was used to assess the integrity of RNA.

### 2.3. Transcriptome Sequencing

The library for sequencing was prepared using the NEBNext Ultra Kit for RNA Library Preparation, with 3 µg of RNA per sample, as per the manufacturer’s instructions. Each sample was assigned an index code to facilitate tracking. The AMPure XP system was utilized to purify the PCR products, and the Agilent Bioanalyzer system was used to evaluate the quality of the library. The indexed samples were clustered using a TruSeq PE Cluster Kit. Throughout the sequencing procedure, 125 bp/150 bp paired-end reads were generated using an Illumina HiSeq platform.

### 2.4. Exosome RNA Analysis

Total RNA was amplified and labeled using a commercial low-input, quick-amp labeling kit, following the manufacturer’s guidelines. The labeled cRNA was then purified using the RNeasy mini kit. Scanning of the slides was carried out on an Agilent Microarray Scanner at a 3 μm resolution, employing the green dye channel, with the photomultiplier tube (PMT) set at 100% and a 20-bit depth setting. Software for feature extraction was used to extract data. Normalization of the raw data was achieved using the quantile algorithm, and the data underwent cleaning to eliminate adapter sequences, poly-N stretches, and reads of low-quality.

### 2.5. Analysis of Differential Gene Expression

The limma R package (version 3.56.2) was employed to conduct differential expression analysis between two conditions. Adjustments to the *p*-values were made using the Benjamini and Hochberg method. Significant differential expression of genes was determined by applying the thresholds of an adjusted *p*-value ≤ 0.05 and an absolute fold change ≥ 2.

### 2.6. Analysis of Gene Ontology and Pathways for the Gene Set

Using the clusterProfiler R package, functional enrichment analyses examined involvement in Gene Ontology (GO) categories—biological processes (BPs), molecular functions (MFs), cellular components (CCs)—and Kyoto Encyclopedia of Genes and Genomes (KEGG) pathways. The terms and pathways with an adjusted *p*-value ≤ 0.05 were considered significant. Visual representations were generated using the ggplot2 R package.

### 2.7. Enrichment of Joint Pathways for the Integrated Analysis of Gene and Metabolomics Datasets

The MetaboAnalyst software’s Joint Pathway Analysis module (www.metaboanalyst.ca) was used to perform an integrated analysis of transcriptomic and metabolic data. This approach combined metabolic datasets with a significance threshold of *p*-value < 0.05 and transcriptomic datasets with a false discovery rate (FDR) < 0.05 and a fold change > 2. Official gene symbols and compound names, accompanied by their log2 fold changes, were uploaded to assess the significance of individual molecules within the networks. For the analysis, key parameters were established, including the use of metabolic pathways from the pathway database, the application of the hypergeometric test for enrichment analysis, the measurement of degree centrality for topology, and the implementation of a combined queries method for integration. Pathways with a *p*-value < 0.05 and an impact score > 0.2 were considered significant [18].

### 2.8. Network Construction and Analysis

The TargetScan tool was utilized to predict the binding sites of miRNAs, circRNAs, and mRNAs. Based on these predictions, circRNA-miRNA-mRNA networks were constructed using Cytoscape. In these networks, nodes represent circRNAs, miRNAs, and mRNAs, and edges illustrate the interaction relationships among them.

### 2.9. SNP and AS Analysis

SNP calling was executed using GATK2 (version 3.7), and SNPs were subsequently annotated with SnpEff software(version 5.2c). Analysis of alternative splicing events, such as skipped exons (SE), retained introns (RI), mutually exclusive exons (MXE), and variation in 5′ and 3′ splice sites (A5SS and A3SS), was conducted using rMATS (version 3.2.5). Alternative splicing is essential for controlling the expression of genes and increasing protein diversity.

## 3. Results

### 3.1. Long-Term Exposure to Low-Dose BaP Significantly Altered Gene Expressions in HCC Cells

**Figure 1:**
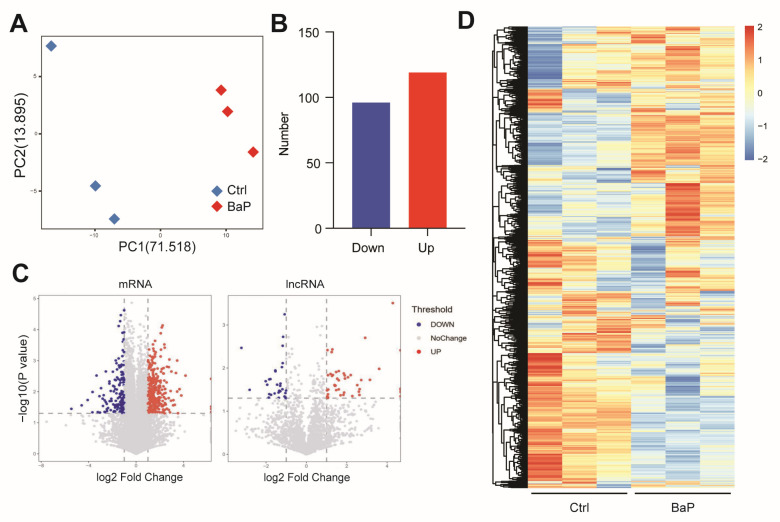
Chronic exposure to BaP caused significant differences in gene expression profiles of HCC cell lines. (**A**) Principal Component Analysis (PCA) plot showing the distribution of samples with BaP treatment (red diamonds) and controls (blue diamonds). (**B**) Bar graph showing the number of genes upregulated and downregulated after BaP treatment. (**C**) Volcano plots showing the effect of BaP treatment on mRNA (left) and lncRNA (right) expression. (**D**) Heatmap comparing gene expression differences between control and BaP groups.

Previously we have reported BaP-exposed HCC cell models by continuous exposure of BaP for one month, which revealed the promoting effect of BaP on HCC metastasis [16]. To further investigate how long-term, low-dose BaP exposure promotes metastasis of HCC cells, we analyzed the RNA of human-derived HCC cells and control cells exposed to long-term, low-dose BaP. Transcriptome sequencing results showed that BaP exposure significantly altered the gene expression pattern of HCC cells. The PCA results showed that BaP exposure caused a significant shift in the gene expression profile of HCC cells (Figure 1A). Differential gene analysis revealed that the number of genes significantly upregulated by BaP exceeded those significantly downregulated (Figure 1B), and most of these differential genes were mRNAs but also included non-coding RNAs such as lncRNAs (Figure 1C). The gene expression heatmap also showed that BaP exposure triggered significant gene expression differences (Figure 1D).

### 3.2. BaP Exposure Significantly Affects Metastasis-Related Gene Expressions in HCC Cells

**Figure 2:**
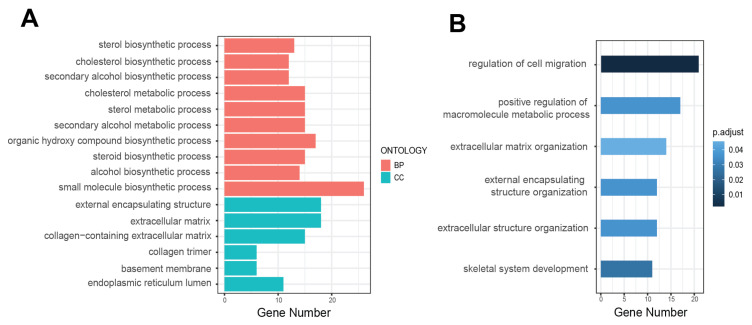
BaP exposure significantly affects metastasis-related processes in HCC cells. (**A**) Bar chart of GO categories for differentially expressed genes, with red indicating biological processes and blue for cellular components. (**B**) Bar chart of GO analysis for upregulated genes, with color gradient representing different adjusted *p*-value.

Further enrichment analysis of differential genes induced by BaP was performed. The results of GO BP revealed that the genes differentially expressed induced by BaP were concentrated in the process related to steroidal lipid metabolism, including steroid biosynthetic process, cholesterol biosynthetic process, and secondary alcohol biosynthetic process (Figure 2A), which are crucial for the composition and function of cell membranes and play significant roles in tumor metabolism and progression. The results of GO CC indicated that BaP caused changes in pathways related to tumor metastasis such as cell migration and invasion, including the extracellular matrix, external encapsulating structure, and basement membrane (Figure 2A). These components play key roles in intercellular signaling and maintaining cell structure, which are crucial for the processes of tumor migration and invasion.

Meanwhile, functional analysis of the upregulated differential genes induced by BaP revealed significant enrichment in pathways related to cell migration, extracellular matrix, and other aspects of cancer metastasis and the tumor microenvironment, specifically, metastasis-related pathways including the regulation of cell migration, extracellular matrix organization, and extracellular structure organization (Figure 2B). The organization of the extracellular matrix (ECM) is essential for providing the structural framework that sup-ports tissue architecture. Alterations in the ECM can facilitate tumor cell invasion by creating paths through which cancer cells can migrate. Similarly, the organization of extracellular structures, including basement membranes and interstitial matrices, plays a role in maintaining tissue integrity and influencing cell behavior, which can promote metastasis. The significant enrichment of these pathways and processes is consistent with our previous finding that long-term, low-dose BaP exposure enhances the migratory and invasive abilities of tumor cells [16].

### 3.3. Effects of BaP Exposure on Metabolic Processes and Key Pathways in HCC Cells

**Figure 3:**
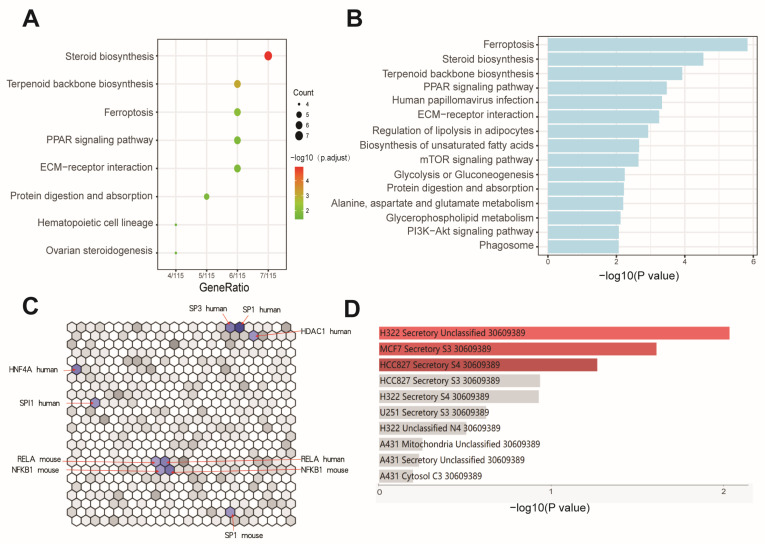
Effects of BaP exposure on metabolic processes and key pathways in HCC cells. (**A**) Bubble plot indicating the enrichment of differentially expressed genes in various biological pathways, with bubble size reflecting gene count and color intensity showing the significance of the adjusted *p*-value. (**B**) Bar chart of pathway enrichment analysis for differentially expressed metabolites and genes, with bar length indicating the significance of each pathway (−log10(*p* value)). (**C**) Network analysis using the TRRUST Transcription Factors database. (**D**) Subcellular localization significance analysis of genes related to cellular secretory proteins, represented by −log10(*p* value).

KEGG enrichment analysis also reconfirmed that BaP affects processes such as steroidal lipid metabolism and extracellular matrix, while significant enrichment of iron death processes was found. This correlates with the significant effect of BaP on lipid metabolism in HCC cells, where BaP exposure may lead to lipid peroxidation and activation of the ferroptosis, which in turn disrupts the homeostasis of intracellular lipid metabolites. We therefore utilized the available metabolomic data of BaP-exposed cells to perform a combined analysis of differential genes and metabolites. The analysis not only highlighted ferroptosis, steroid lipid metabolism, and extracellular matrix processes again but also revealed significant enrichment of signaling pathways closely related to tumor metastasis, such as PI3K-AKT and mTOR, suggesting that these pathways are likely involved in BaP promotion of tumor metastasis. Meanwhile, the signaling pathway of PPAR, as a key endogenous receptor for lipid metabolites, was identified in both KEGG analysis and differential gene/metabolite combination analysis results (Figure 3A,B), which also further confirmed the changes in lipid metabolism. The effect of BaP on the activity of cellular transcription factors was also investigated, and the results showed that BaP mainly influenced the functionality of transcription regulators, such as NF-κB, SP1, and HNF4A (Figure 3C), which was in line with the finding that the NF-κB pathway mediated the toxic effect of BaP in our previous experimental results. In addition, cellular localization of differential genes showed that genes related to cellular secretory proteins were significantly enriched (Figure 3D), suggesting that BaP may promote tumor metastasis by altering gene expression in HCC cells, which in turn affects exosomal contents.

### 3.4. Effect of BaP Exposure on the RNA Profiling in Exosome of HCC Cells

**Figure 4:**
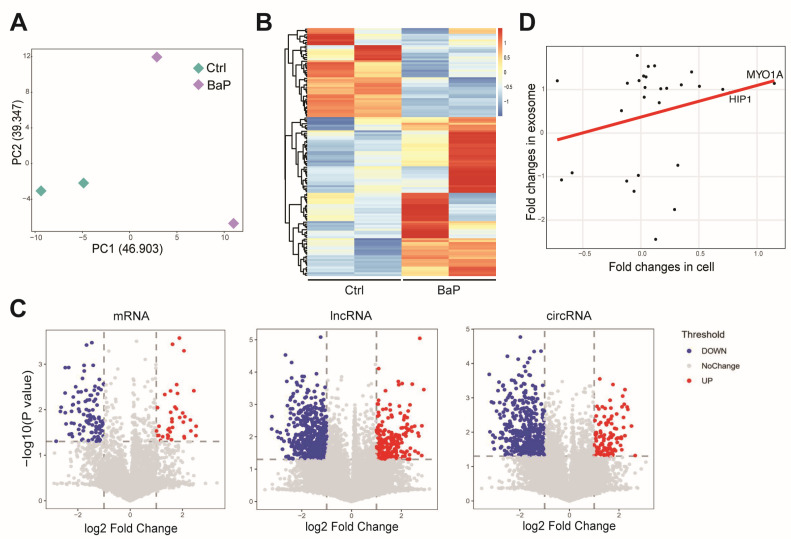
Effect of long-term BaP exposure on exosome-carrying RNAs in HCC cells. (**A**) PCA plot demonstrating the impact of long-term, low-dose BaP exposure on the RNA composition of exosomes in HCC cells. (**B**) Heatmap revealing the changes in the expression levels of mRNAs, lncRNAs, and circRNAs in the exosomes of HCC cells due to BaP treatment. (**C**) Volcano plots comparing the impact of BaP treatment on the expression of different types of RNAs in the exosomes of HCC cells. (**D**) Scatter plot comparing gene expression changes in HCC cells and exosomes, revealing the correlation of gene expression inside and outside the cells.

To further assess the effect of long-term, low-dose BaP exposure on the exosomes of HCC cells, we used microarray technology to detect mRNA, lncRNA, and circRNA content in exosomes of BaP-exposed and control HCC cells. We found that BaP exposure significantly altered the RNA composition in exosomes (Figure 4A,B) and that these changes also differed more among biological replicates, suggesting that imbalance of the exosome system as a result of BaP exposure may promote tumor metastasis. Examination of various RNA types revealed that BaP exposure resulted in differential expression levels of mRNAs, lncRNAs, and circRNAs in the exosomes of HCC cells to varying degrees (Figure 4C). Some RNAs (e.g., MYO1A and HIP1) showed similar trends of changes in cells and exosomes, whereas the exosomal content of other RNAs increased but was not related to the intracellular expression levels (Figure 4D). This suggests that BaP not only affects the composition of RNAs carried by the exosomes of HCC cells by altering their gene expression levels but also more likely alters the components of the exosomes of HCC by affecting the process of transport and packaging of RNAs into the exosomes.

### 3.5. BaP-Induced Exosomal circRNA-miRNA-mRNA Interaction Network in HCC Cells

**Figure 5:**
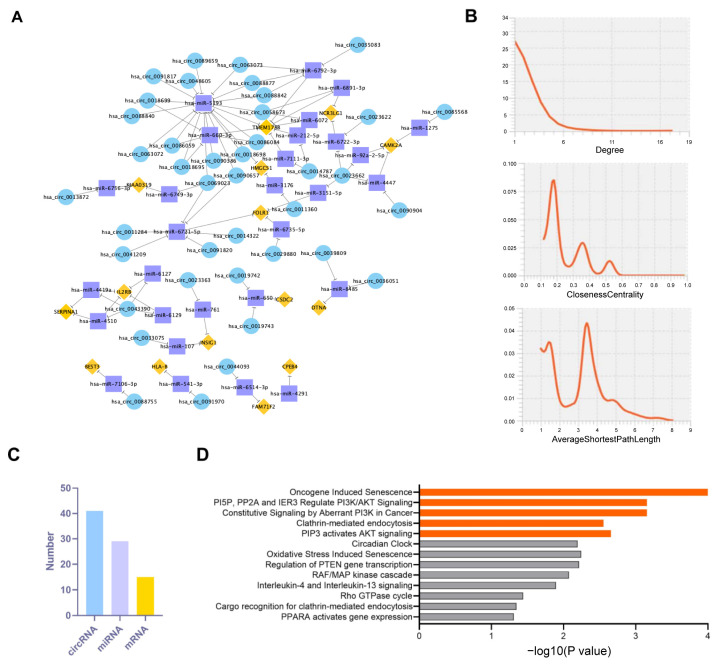
Exosomal circRNA-miRNA-mRNA interaction network analysis in HCC cells. (**A**) Significant alterations in the circRNA-miRNA-mRNA interaction network within exosomes of HCC cells after BaP exposure. (**B**) Distribution of degree, closeness centrality, and average shortest path length in the network. (**C**) Bar chart reflecting the number of differential circRNAs caused by BaP, and the count of their target miRNAs and mRNAs. (**D**) Enrichment analysis histogram showing the enrichment results of miRNA carried by HCC cells.

To gain a deeper understanding of how chronic exposure to low-dose BaP affects the gene and non-coding RNA content in the exosomes of HCC cells, we mapped the circRNA-miRNA-mRNA interactions network, in which BaP exposure induced significant alterations in the exosomes of HCC cells based on the relationship between the MREs contained in circRNAs and the target mRNAs of the corresponding miRNAs (Figure 5A,B). We found that BaP exposure could affect miRNAs in recipient cells by inducing circRNAs in HCC exosomes, thereby altering mRNA levels. Although the number of differential circRNAs caused by BaP was high, the number of its target miRNAs and mRNAs was relatively low (Figure 5C), suggesting a specific concentration of biological effects. miRNA enrichment analysis showed that HCC cells carried miRNAs, with a concentration of miRNAs acting on active pathways such as PI3K/AKT, PTEN, and cellular senescence (Figure 5D), which suggests that BaP regulates the tumor microenvironment and influences the cellular state through exosomes, thus providing the basis for tumor cell metastasis.

### 3.6. BaP-Induced Gene Mutation and Genomic Instability in HCC Cells

**Figure 6:**
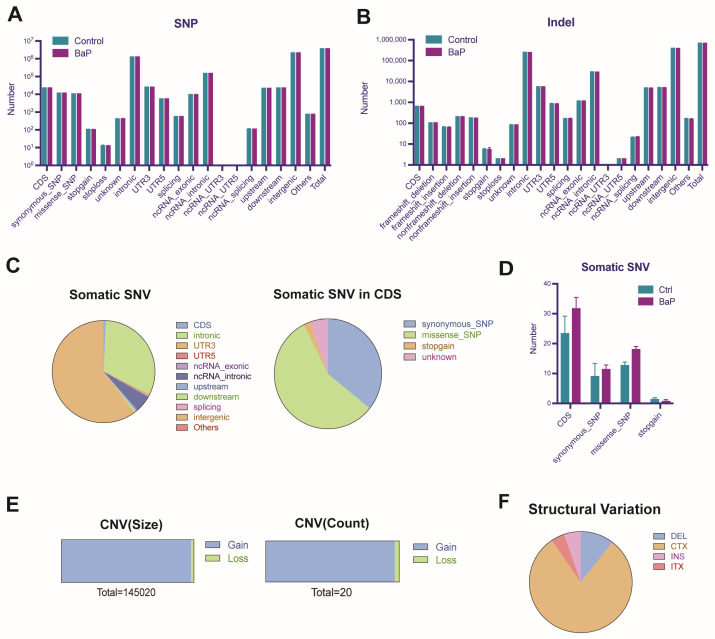
Gene mutation analysis of HCC cells induced by BaP exposure. (**A**) Bar chart showing the changes in the number of known single nucleotide polymorphisms (SNPs) in HCC cells before and after long-term, low-dose exposure to BaP. (**B**) Bar chart demonstrating the effect of long-term, low-dose BaP exposure on insertion–deletion (Indel) variants in HCC cells. (**C**) Pie chart reflecting the distribution of types of somatic single nucleotide variations (SNVs) in HCC cells caused by BaP exposure. (**D**) Bar chart indicating the increase in missense mutations in the coding regions (CDS) of HCC cells due to BaP exposure. (**E**) Sidebar graph showing gene copy number variations in HCC cells due to BaP exposure. (**F**) Pie chart revealing the impact of BaP exposure on chromosomal structural variations in HCC cells.

Finally, considering the mutagenic properties of BaP, we explored the potential impacts of prolonged low-dose exposure to BaP on gene mutations in HCC cells. Whole genome sequencing showed that BaP did not significantly alter the number of known single nucleotide (SNP) and insertion–deletion (Indel) variants (Figure 6A,B), which may be related to the low exposure dose of BaP. However, the increase in de novo mutation sites, especially missense mutations in the coding region (CDS), implied that BaP might cause abnormal protein function (Figure 6C,D). The vast majority of the abnormalities in the gene copy number caused by BaP exposure to HCC cells were copy number increases, and the chromosomal structural variations were mainly translocations, with a few losses, insertions, and inversions (Figure 6E,F), suggesting that BaP affects genomic stability.

## 4. Discussion

The most common type of primary liver cancer, HCC, is the fourth largest cause of cancer-related deaths globally [19]. The process of BaP-induced hepatocarcinogenesis involves gene mutations, chromosomal aberrations, and epigenetic alterations. Research has demonstrated that treatment with BaP enhances the invasive properties of lung cancer cells in vitro [20], increases the migration and invasion of lung cancer cells [21], boosts the invasive potential of human breast cancer cells [22], and increases the in vitro migration and invasive ability of hepatocellular carcinoma Hep-G2 cells [23]. Our previous study also observed that chronic exposure to BaP promoted the migration and invasion of HCC cells both in vitro and in vivo [16]. These suggest a broader, oncogenic role of BaP across different tissue origins, reinforcing the need for stringent controls and monitoring of environmental BaP exposure.

The PI3K-AKT-mTOR pathway plays a vital role in promoting tumorigenesis and sustaining tumor growth, making it one of the most commonly disrupted pathways in human cancers [24]. AKT activation starts downstream signaling, such as the mTOR pathway, which increases protein synthesis by phosphorylating S6 kinases and 4E-BP. This process facilitates cell growth and proliferation [25]. In hepatocarcinogenesis, the PI3K/AKT/mTOR pathway often shows dysregulation, and PTEN, a negative regulator of this pathway, typically acts as a tumor suppressor. Low PTEN expression correlates with intrahepatic metastasis of HCC and poor prognosis [26]. In our study, we enriched miRNAs and found that the miRNA action pathways carried out by HCC cells were mainly focused on the active pathways such as PI3K/AKT and PTEN, suggesting the potential involvement of exosomes in metastasis.

BPDE, the primary active metabolite of BaP, is highly reactive and can directly bind to DNA, forming adducts that cause DNA damage and mutations [7]. Additionally, BPDE activates multiple cell signaling pathways, induces the production of inflammatory factors, and plays a role in HCC progression. Although BaP can be metabolized into BPDE in HCC cells, the extent to which BPDE mediates the effects of BaP on HCC progression remains unclear. In our study, although we mainly focused on the effects of BaP on HCC cells, the role of its metabolite BPDE is equally important. But, whether it is BaP itself or its metabolite BPDE, the ultimate effect is due to exposure to BaP.

Exosomes are a crucial transduction mediator of material exchange and information exchange between tumor cells and the microenvironment. Tumor exosomes are vesicles secreted by tumor cells into the extracellular environment, which interact with the microenvironment by carrying a variety of active molecules to remodel the microenvironment suitable for tumorigenesis and development [27]. Exosomes can regulate mesenchymal stromal cells in the tumor microenvironment to generate a microenvironment in which cancer cells are resistant to radiotherapy [28], and they can also recruit and domesticate cancer-promoting immune cells, shaping a microenvironment conducive to the immune escape of cancer cells [29]. Numerous studies have demonstrated that exosomes from different types of tumors, including HCC, can shape the local microenvironment suitable for tumor metastasis by targeting and activating tumor-associated fibroblasts, recruiting bone-marrow-derived macrophages, modulating endothelial cells and other microenvironmental cells, which in turn promotes invasive metastasis of tumors [30,31,32,33,34]. BaP activates relevant signaling pathways by altering gene expression patterns and exosomal RNA components in HCC cells, which in turn enhances the migration and invasion of tumor cells. These findings provide new insights into how BaP promotes tumor metastasis through multiple mechanisms at the molecular level. In particular, the effects of BaP on exosomal RNA composition and the regulation of transcription factor activity reveal that it may affect the tumor microenvironment and intercellular communication through atypical pathways, providing new mechanisms for tumor metastasis. In addition, the genomic stability changes induced by BaP, especially the increase in missense mutations, suggest that it might further promote tumor progression by directly affecting gene coding and protein function. These results not only improve our understanding of BaP carcinogenesis but also provide important molecular targets for future exploration of BaP-induced HCC metastasis.

Our study has some limitations. The in vitro approach may not fully replicate the complex in vivo environment. Additionally, the specific HCC cell lines used may not represent the heterogeneity of patient tumors. BPDE was not assessed in this study, and future research should include BPDE to explore its specific mechanisms of action. Further in vivo studies and increased replication are needed to confirm these findings and better understand BaP’s impact on HCC progression.

## 5. Conclusions

The current study showed that prolonged low-dose exposure to BaP not only altered the gene expression pattern of HCC cells, especially the genes related to steroidal lipid metabolism and cell migration, but also induced significant changes in the RNA components in the exosomes of HCC cells, including the expression of mRNA, lncRNA, and circRNA. These changes affected key cancer signaling pathways, such as PI3K/AKT and PTEN, in recipient cells in the tumor microenvironment, laying the foundation for further metastasis of tumor cells. In addition, BaP induced novel mutations in the genomic DNA of HCC cells, which may have aberrant effects on protein function. These findings highlight the role of environmental carcinogens in the development of HCC and point to the potential role of exosomes in tumor metastasis, providing new strategic directions for future hazard prevention and control of BaP toxicity.

## Data Availability

The original contributions presented in this study are included in the article; further inquiries can be directed to the corresponding authors.
